# Global migration of clinical research during the era of trial registration

**DOI:** 10.1371/journal.pone.0192413

**Published:** 2018-02-28

**Authors:** Paul K. Drain, Robert A. Parker, Marion Robine, King K. Holmes

**Affiliations:** 1 Department of Global Health, University of Washington, Seattle, WA, United States of America; 2 Department of Medicine, School of Medicine, University of Washington, Seattle, WA, United States of America; 3 Department of Epidemiology, School of Public Health, University of Washington, Seattle, WA, United States of America; 4 Medical Practice Evaluation Center, Department of Medicine, Massachusetts General Hospital, Boston, MA, United States of America; 5 Division of Infectious Diseases, Massachusetts General Hospital, Harvard Medical School, Boston, MA, United States of America; 6 Biostatistics Center, Department of Medicine, Massachusetts General Hospital, Boston, MA, United States of America; University of British Columbia, CANADA

## Abstract

**Background:**

Since the site of human subjects research has public health, regulatory, ethical, economic, and social implications, we sought to determine the global distribution and migration of clinical research using an open-access trial registry.

**Methods:**

We obtained individual clinical trial data including location of trial sites, dates of operation, funding source (United States government, pharmaceutical industry, or organization), and clinical study phase (1, 1/2, 2, 2/3, or 3) from ClinicalTrials.gov. We used the World Bank's classification of each country's economic development status ["High Income and a Member of the Organization for Economic Co-operation and Development (OECD)", "High Income and Non-Member of the OECD", "Upper-Middle Income", "Lower-Middle Income", or "Low Income"] and United Nations Populations Division data for country-specific population estimates. We analyzed data from calendar year 2006 through 2012 by number of clinical trial sites, cumulative trial site-years, trial density (trial site-years/106 population), and annual growth rate (%) for each country, and by development category, funding source, and clinical study phase.

**Results:**

Over a 7-year period, 89,647 clinical trials operated 784,585 trial sites in 175 countries, contributing 2,443,850 trial site-years. Among those, 652,200 trial sites (83%) were in 25 high-income OECD countries, while 37,195 sites (5%) were in 91 lower-middle or low-income countries. Trial density (trial site-years/106 population) was 540 in the United States, 202 among other high-income OECD countries (excluding the United States), 81 among high-income non-OECD countries, 41 among upper-middle income countries, 5 among lower-middle income countries, and 2 among low-income countries. Annual compound growth rate was positive (ranging from 0.8% among low-income countries to 14.7% among lower-middle income countries) among all economic groups, except the United States (-0.5%). Overall, 29,191 trials (33%) were funded by industry, 4,059 (5%) were funded by the United States government, and 56,397 (63%) were funded by organizations. Countries with emerging economies (low- and middle-income) operated 19% of phase 3 trial sites, as compared to only 6% of phase 1 trial sites.

**Conclusion:**

Human clinical research remains concentrated in high-income countries, but operational clinical trial sites, particularly for phase 3 trials, may be migrating to low- and middle-income countries with emerging economies.

## Introduction

Research involving human subjects has evolved over millennia–from an observational study in the Old Testament [1] to the first randomized clinical trial in 1944 [2]. Historically, clinical research has been financed by pharmaceutical companies and conducted in affluent regions, but rising drug development expenditures have increased demand for faster and cheaper trial results [3]. Currently, over 3,000 clinical research organizations (CROs) operate worldwide with total combined market revenues exceeding $21 billion [4–6]. Since researchers, including CROs, use global networks to accelerate patient recruitment and reduce costs, we sought to quantify the global distribution and migration of clinical research [4,6,7].

Since the ethical conduct of international clinical research remain a prominent concern, we sought to perform a comprehensive assessment of the global migration of clinical research. During the 1990s, underreported and unethical human studies led to calls for research transparency through clinical trial registration [8,9]. The United States Food and Drug Administration (FDA) mandated the creation of an open-access clinical trial registry, ClinicalTrials.gov [9], and the World Health Organization (WHO) established the International Clinical Trials Registry Platform [10]. In 2005, the International Committee of Medical Journal Editors (ICMJE) required registration in order to publish results, and compliance to trial registration accelerated quickly [11–14].

We expand upon our prior analyses of the WHO International Clinical Trials Registry Platform [15], which is a more comprehensive database, by using more detailed operational data in ClinicalTrials.gov and new methodological contributions to estimate the global conduct of clinical research. By using the ClinicalTrials.gov data, we have been able to integrate analyses on study site locations, duration of trial site operation, study phase, and funding source. We developed a metric—clinical trial site-years—to more accurately compare research activity across countries. By presenting a detailed account of the conduct of clinical research, we may better understand the global implications of advancing medicine while maintaining protection for human research subjects.

## Methods

### Primary data source

Our primary data source was ClinicalTrials.gov [9], created in 1999 by the National Institutes of Health’s Library of Medicine as the first open-access clinical trial registry [11,13,16]. Since its inception, ClinicalTrials.gov has remained the primary registry of human clinical research worldwide [9]. When the ICMJE required trial registration in 2005, ClinicalTrials.gov was the only acceptable registry [11,13,16]. By 2008, the vast majority of published clinical trials were registered in ClinicalTrials.gov [17]. While other open-access registries have been created [15], ClinicalTrials.gov has remained the largest and most comprehensive clinical trial registry worldwide [9].

We obtained the Aggregate Analysis of Clinical Trials (AACT) dataset, a reformatted version of publicly available data, from the Clinical Trials Transformation Initiative on 5 May 2014 [18]. The dataset contained available data through 27 September 2013. We limited our analysis to operational clinical trials through the end of 2012 to have a sufficient reporting grace period. Since one clinical trial may have multiple trial sites, we obtained data on individual trial site locations to better represent the volume of clinical research activity in different countries. Trial site opening and closing dates were not available, so we assumed each site operated for the entirety of the associated trial for assessing time trends in research activity. This new metric, trial site-years, better reflects a comparison of research activity across locations.

The dataset included primary funding source and study phase for each clinical trial. Funding source was categorized as United States government-sponsored (“National Institutes of Health” or “Other United States Federal Agency”), industry-sponsored (“pharmaceutical companies”), or organization-sponsored (“Individual/University/Organizations, including community-based organizations”). Clinical study phase was based on the United States FDA’s classification system (Phase 1 defined as “conducted with healthy volunteers and emphasize safety”; Phase 2 defined as “preliminary data on effectiveness”; Phase 3 defined as “information about safety and effectiveness by studying different populations and different dosages”). We combined Phase 0 studies (defined as “exploratory studies involving very limited human exposure to the drug”) with Phase 1 studies as both reflect early clinical trials. We excluded phase 4 studies [defined as “studies occurring after FDA has approved a drug for marketing”; 10,778 (11%) of 100,425 studies], since post-marketing studies are confounded by geographic disease burden and drug affordability. In addition, including post-marketing surveillance studies, may have detracted from our goal of understand the global migration of clinical and translational research. We retained studies with phase coded as N/A [42,319 (42%) of 100,425 studies] for the analysis of study sites. Studies with multiple phases (1/2 or 2/3) were reported separately.

We excluded trial site records without a country code (N = 6) and records from countries or regions without an economic classification by the World Bank [regions of France (Martinique, Guadeloupe, and Reunion; N = 19), Former Yugoslavia (N = 5), Former Serbia and Montenegro (N = 87), Kosovo (N = 1), Palestinian Territories (N = 4), and Holy See (N = 1)]. Puerto Rico and Taiwan were represented as independent entities. We excluded 465,871 trial sites occurring only before the year 2006, only after the year 2012, or only in phase 4 studies.

### Other data sources

We classified each country’s economic development status according to World Bank categories [“High Income and a Member of the Organization for Economic Co-operation and Development (OECD)”, “High Income and Non-Member of the OECD”, “Upper-Middle Income”, “Lower-Middle Income”, or “Low Income”] in the World Development Report for 2006, which was the first year of our study period (S1 Table) [19]. We obtained each country’s estimated total population for the year 2006 from United Nations Populations Division [20]. We obtained populations for Taiwan from International Monetary Fund [21] and Netherlands Antilles from United Nations statistics [22].

### Patient involvement

Although we did not include active patient engagement in setting the research agenda for this article, all participants of human clinical research trials worldwide may be impacted by the implications of these findings.

### Statistical analyses

We separated the United States from other high-income OECD countries in all analyses, due to its high proportion of clinical trials. We used the term “emerging economies” to refer to upper-middle income, lower-middle income, and low-income countries. We used the term “trial site-years” to represent an operational clinical trial site during one calendar year, and used this measure to calculate average annual density and compounded annual growth rate. Trial density was calculated as the annual number of operating trial site-years per million people. Since the total number of registered clinical trial sites remained low during the first 7 calendar years (1999–2005) and increased by 22% from 2005 to 2006, we restricted all analyses to the 2006–2012 calendar years. In addition, excluding the underreporting period before and through 2005 prevented spuriously high estimates for annual growth rates. We independently ranked the top 20 countries by number of trial sites, average annual trial-site density, and annual compound growth rate. The growth rate rank list was limited to countries with ≥5 sites for each year and ≥100 total trial sites between 2006 and 2012 to prevent spuriously high rates due to small relative changes. We excluded countries with few clinical trials, since very small changes in number of clinical trials or trial sites spuriously reflected very large growth in clinical research, but only a small change in absolute numbers of clinical trials. To calculate compound annual growth, we used linear regression on the log of trial sites per year and 95% confidence intervals were calculated based on the standard error of the regression coefficient, converted back to percent growth. We calculated the annual proportion of all trials by funding source and by study phase. All calculations were performed using SAS version 9.4 (Cary, NC, USA).

## Results

**Fig 1:**
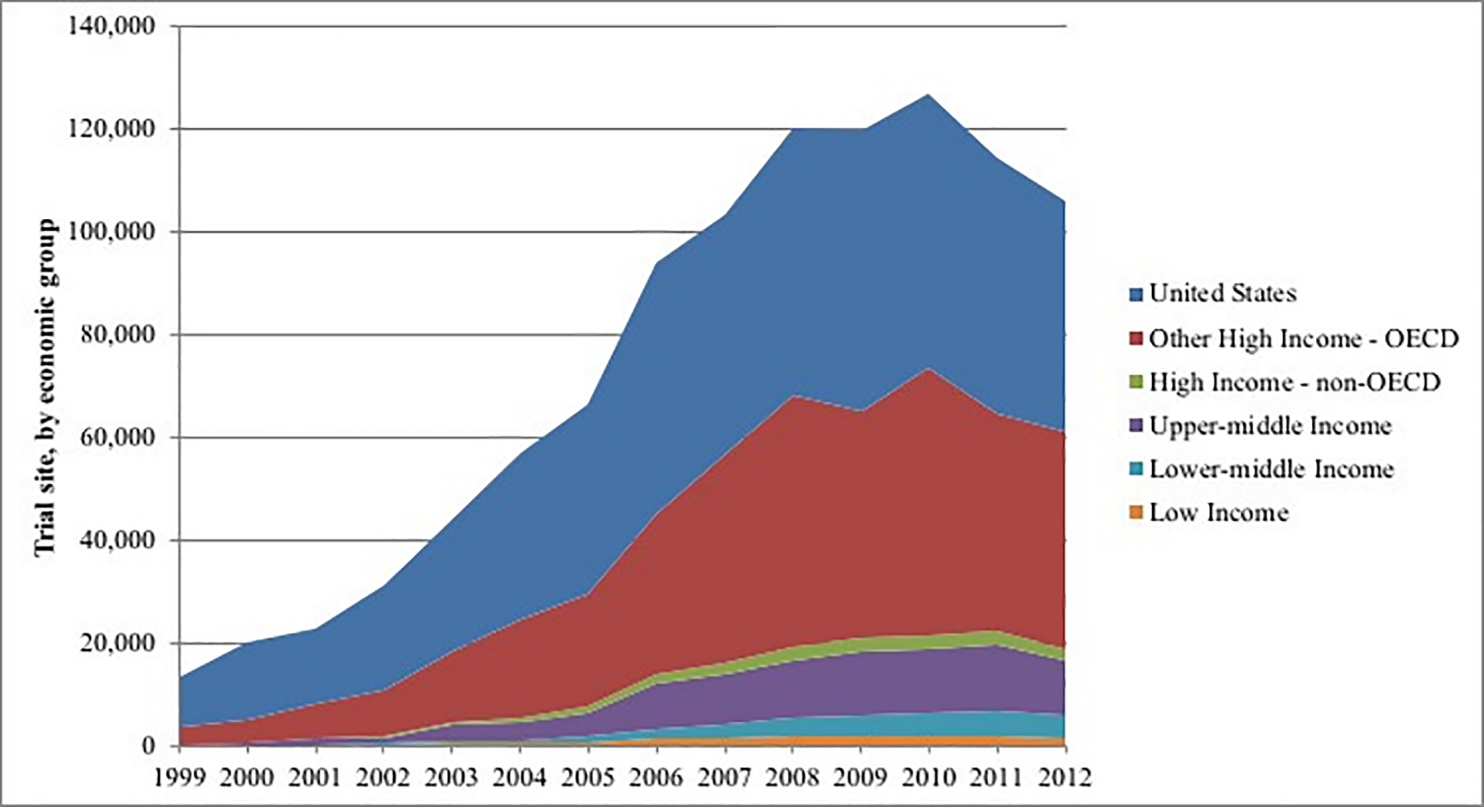
Total annual number of registered clinical trial sites beginning a study by World Bank economic development category. Data for the United States and other high-income OECD countries are displayed separately. Organization-sponsored funding sources included individuals, universities, foundations, and community-based organizations.

Over the 7-year period, 89,647 clinical trials operated 784,585 trial sites in 175 countries, contributing 2,443,850 trial site-years of observation (Table 1). Among those, 652,200 trial sites (83%) and 2,052,126 trial site-years (84%) were operating in the 25 high-income OECD countries. Conversely, the 125 countries with emerging economies accounted for 115,684 trial sites (15%) and 342,053 trial site-years (14%). As expected, the distribution of clinical trial sites was very heterogeneous between economic development categories (Fig 1).

**Table 1:** Clinical trials by economic development status, 2006–2012.

Development Status	Number of Countries	Trial Sites N (%)	Trial Site-Years N (%)	Population (x 10^3^)	Average Annual Trial-Site-Year Density (/10^6^ people)	Trial-Site-Year Density in 2012 (/10^6^ people)	Annual Growth Rate (95% CI)
United States	1	350,592 (45)	1,138,447 (47)	300,943	540	826	-0.5 (-3.3, 2.4)
High Income–OECD (excluding U.S.)	24	301,608 (38)	913,679 (37)	645,460	202	306	3.9 (-2.1, 10.2)
High Income–non-OECD	25	16,701 (2)	49,671 (2)	87,544	81	126	6.1 (1.4, 11.0)
Upper-middle Income	34	78,489 (10)	235,589 (10)	817,979	41	62	4.6 (0.2, 9.1)
Lower-middle Income	45	26,531 (3)	74,080 (3)	2,286,807	5	8	14.7 (9.0, 20.7)
Low Income	46	10,664 (1)	32,384 (1)	2,382,899	2	3	0.8 (-5.7, 7.7)
**Total**	**175**	**784,585 (100)**	**2,443,850 (100)**	**6,521,631**	**54**	**82**	**2.2 (-1.6, 6.2)**

CI–Confidence Interval, OECD—Organization for Economic Co-operation and Development.

Since 2006, the average annual number of trial sites was 112,084, ranging from 93,975 (2006) to 127,024 (2010). Over the seven-year period, the global proportion of clinical trial sites in the United States decreased from 52% to 43%, while the proportion among other high-income OECD countries increased from 33% to 40%. The proportion of trial sites also increased among lower-middle income countries (2% to 4%) from 2006 to 2012.

Fourteen (70%) of the top 20 countries by total number of trial site-years were high-income (13 OECD countries and one non-OECD), and the top 10 countries accounted for 77% of trial site-years (1,883,571 trial site-years) during the 7-year period (S2 Table). The United States operated the most trial site-years during the study period and the most trial sites in the year 2012. In addition, Germany, France, Japan, and Canada also operated >20,000 trial sites between 2006–2012. The countries in the top 20 that were not high-income OECD countries were the Russian Federation, Poland, China, India, Brazil, and Hungary.

### Clinical trial density

The global average annual clinical trial density, which accounts for population size, was 54 trial site-years/10^6^ people (Table 1). The United States had the highest trial density (540 trial site-years/10^6^ people), followed by other high-income OECD countries (202/10^6^) and high-income non-OECD countries (81/10^6^). Countries with upper-middle income (41 trial site-years/10^6^ people), lower-middle income (5/10^6^), and low-income (2/10^6^) had lower densities of clinical trial site-years. This pattern was consistent for the year 2012, and trial site densities varied considerably by economic development status.

Seventeen (85%) of the top 20 countries with the highest density of clinical trial site-years were high-income countries (Table 2). The United States (540 trial site-years/10^6^ population), Belgium (503/10^6^), Israel (392/10^6^), Denmark (361/10^6^), and Canada (356/10^6^) had the highest average annual density. The members in this list that were not high-income countries were Hungary, Slovakia, and Latvia. Notably, some countries, such as Japan, the United Kingdom, and Italy had a large total number of trial sites, but were not among the top 20 countries for trial site density due to a large relative population size.

**Table 2:** Density of clinical trial sites (per million people) by country, ranked by average annual trial density 2006–2012.

Rank	Country	Average Annual Trial Site Year Density* (2006–2012)	Trial Site Year Density* (2012)	Development Status
**1**	United States	540	826	OECD
**2**	Belgium	503	709	OECD
**3**	Israel	392	621	N-OECD
**4**	Denmark	361	534	OECD
**5**	Canada	356	507	OECD
**6**	Czech Republic	344	484	OECD
**7**	Germany	325	482	OECD
**8**	France	311	436	OECD
**9**	Hungary	299	487	UMC
**10**	Estonia	298	475	N-OECD
**11**	Slovakia	289	401	UMC
**12**	Austria	281	403	OECD
**13**	Sweden	253	364	OECD
**14**	Norway	229	326	OECD
**15**	Switzerland	229	337	OECD
**16**	Netherlands	222	337	OECD
**17**	Finland	216	295	OECD
**18**	Australia	215	333	OECD
**19**	Latvia	193	317	UMC
**20**	Spain	192	308	OECD

OECD (Organization for Economic Co-operation and Development). OECD–High-income: OECD; N-OECD–High-income: non-OECD; UMC–Upper-Middle Income.

* Trial site year density was the number of registered clinical trial site-years divided by country population in millions.

### Average annual clinical trial growth rate

Since 2006, the overall annual clinical trial compound growth rate was 2.2% (Table 1). Annual growth rates were highest among lower-middle income countries (14.7%), high-income non-OECD countries (6.1%), and upper-middle income countries (4.6%). Annual growth rates were positive across all economic development strata, except the United States (-0.5%).

In sharp contrast to trial site density, 17 (85%) of the top 20 countries ranked by highest annual growth rate were countries with emerging economies (Table 3). Countries with the highest average annual growth rates were Lebanon (41.9%), Egypt (28.3%), Saudi Arabia (27.4%), Guatemala (27.0%), and China (24.8%). High-income countries on this list included Saudi Arabia, Republic of Korea, and Japan.

**Table 3:** Average annual clinical trial site growth rate by country, ranked by average annual growth rate 2006–2012.

Rank	Country	Annual Growth Rate* (95% CI)	Total Trial Site-years (2006–2012)	Development Status
**1**	Lebanon	41.9 (14.2, 76.2)	283	UMC
**2**	Egypt	28.3 (18.3, 39.0)	591	LMC
**3**	Saudi Arabia	27.4 (5.0, 54.6)	420	N-OECD
**4**	Guatemala	27.0 (10.2, 46.2)	306	LMC
**5**	China	24.8 (19.2, 30.7)	10,681	LMC
**6**	Belarus	21.5 (-9.9, 63.7)	241	LMC
**7**	Korea, Republic of	20.6 (11.6, 30.4)	9,137	OECD
**8**	Kenya	18.7 (2.9, 36.9)	213	LIC
**9**	Colombia	17.6 (7.9, 28.1)	1,579	LMC
**10**	Bosnia and Herzegovina	17.2 (0.3, 36.9)	179	LMC
**11**	Japan	15.5 (4.6, 27.5)	29,052	OECD
**12**	Venezuela	14.9 (-4.8, 38.6)	164	UMC
**13**	Bangladesh	13.9 (1.4, 27.9)	112	LIC
**14**	Uganda	13.5 (2.4, 25.8)	177	LIC
**15**	Turkey	12.7 (2.0, 24.5)	2,679	UMC
**16**	Bulgaria	12.0 (9.4, 14.6)	3,109	UMC
**17**	Panama	11.7 (-2.3, 27.6)	138	UMC
**18**	Vietnam	11.1 (-8.4, 34.8)	220	LIC
**19**	Hungary	10.6 (6.9, 14.5)	7,468	UMC
**20**	Georgia	9.7 (-13.3, 38.7)	235	LMC

CI–Confidence Interval, OECD (Organization for Economic Co-operation and Development). OECD–High-income: OECD; N-OECD–High-income: non-OECD; UMC–Upper-Middle Income; LMC–Lower-Middle Income; LIC–Low Income.

*Due to underreporting before 2005, we ranked countries with ≥5 sites for each year and ≥100 total trial sites between 2006 and 2012 by compounded average annual clinical trial growth rate from 2006–2012.

### Clinical trials by funding source

**Fig 2:**
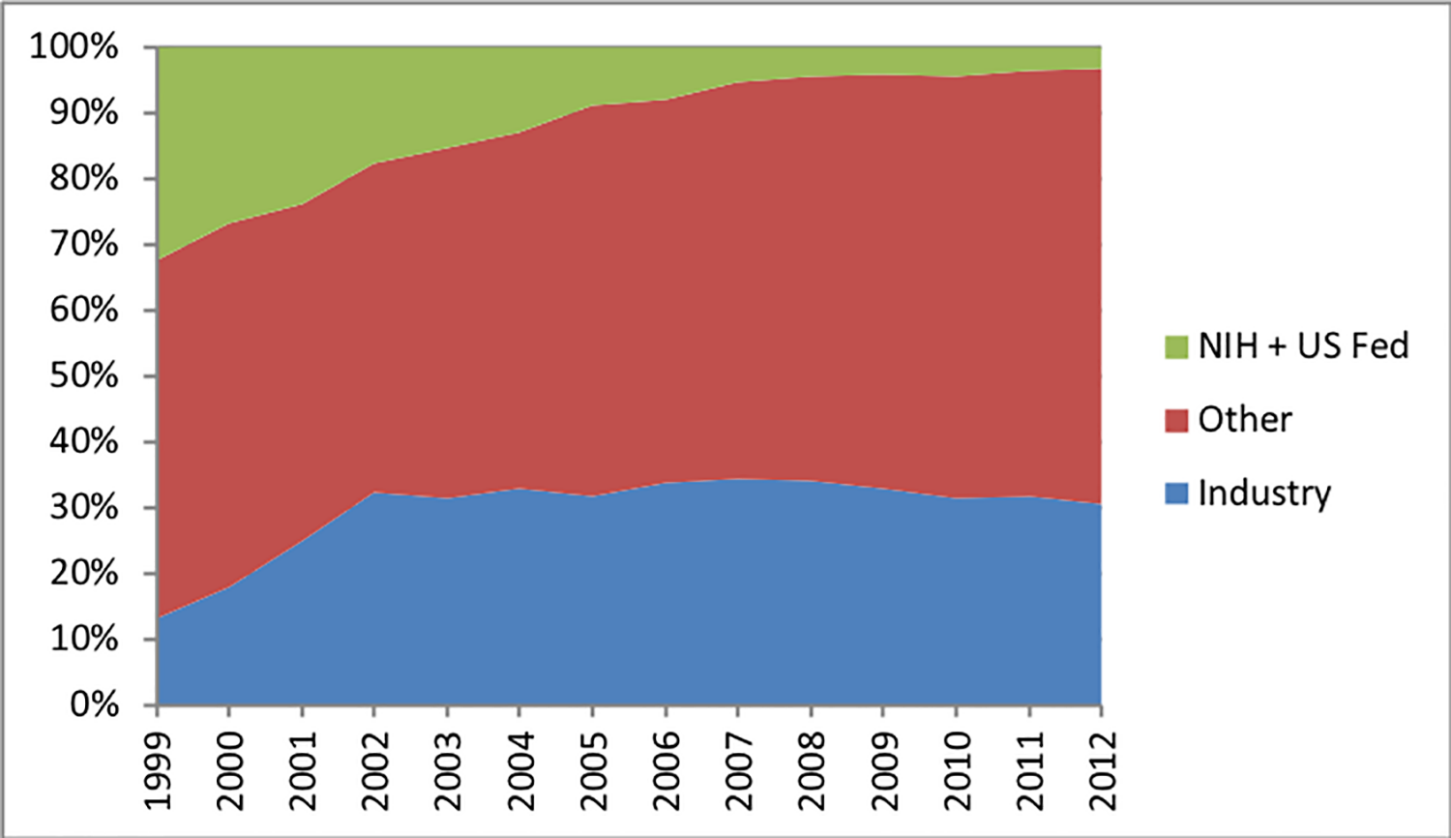
Total annual number of registered clinical trial sites beginning a study by proportion of clinical trials starting by funding source. Data for the United States and other high-income OECD countries are displayed separately. Organization-sponsored funding sources included individuals, universities, foundations, and community-based organizations.

During the 7-year period, 29,191 (33%) trials were funded by the pharmaceutical industry, 4,059 (5%) were funded by the United States government, and 56,397 (63%) were funded by other organizations (Fig 2). Between 2006–2012, trials sponsored by the United States government decreased from 8% in 2006 to 3% in 2012. Clinical trials sponsored by other organizations increased from 5,558 trials (58%) in 2006 to 9,451 trials (66%) in 2012. During the same period, industry sources funded between 35% (year 2007) and 31% (year 2012) of all clinical trial sites.

### Clinical trial site by study phase

**Fig 3:**
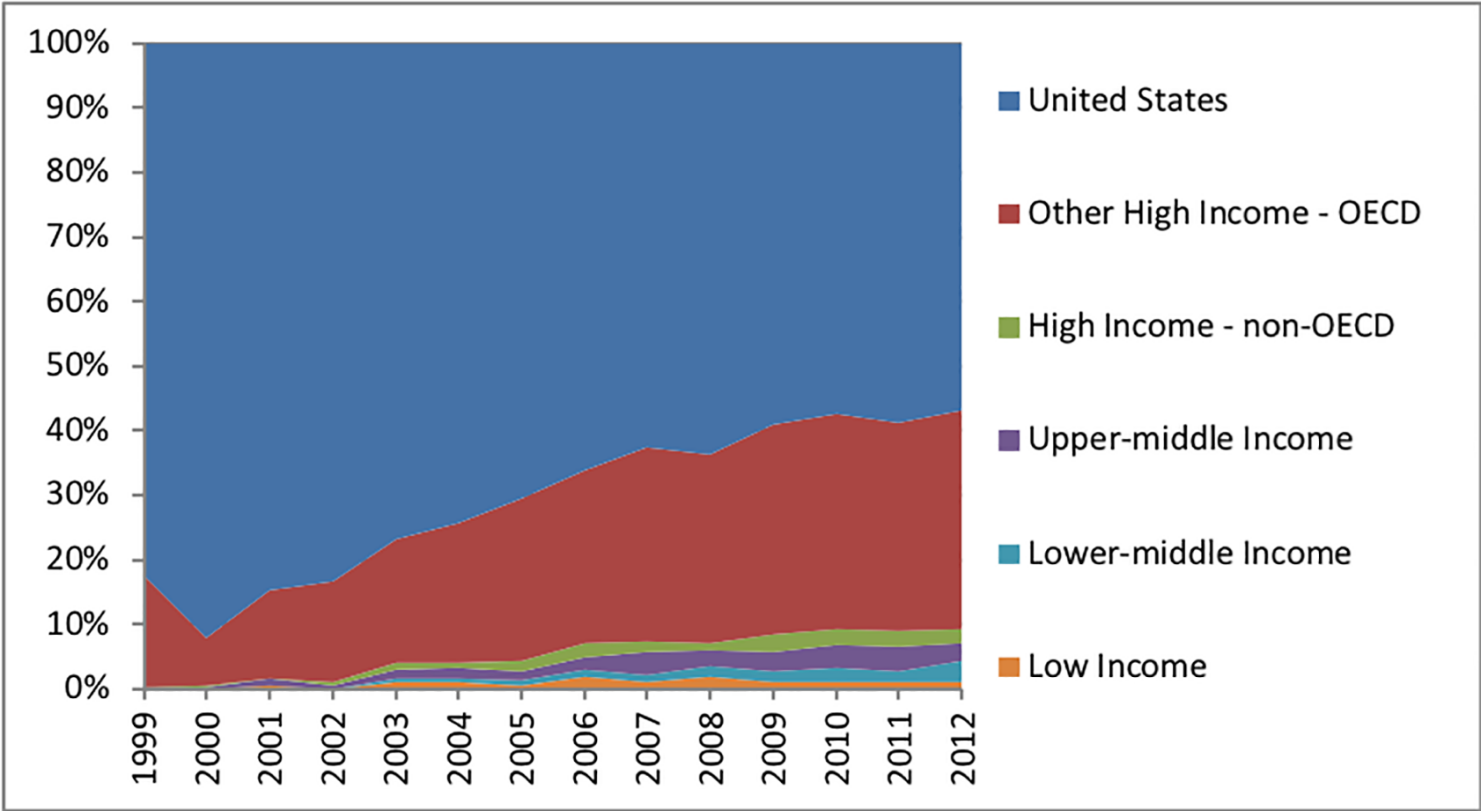
Proportion distribution of registered clinical trials among phase 1 studies by World Bank economic development category. Data for the United States and other high-income OECD countries are displayed separately.

**Fig 4:**
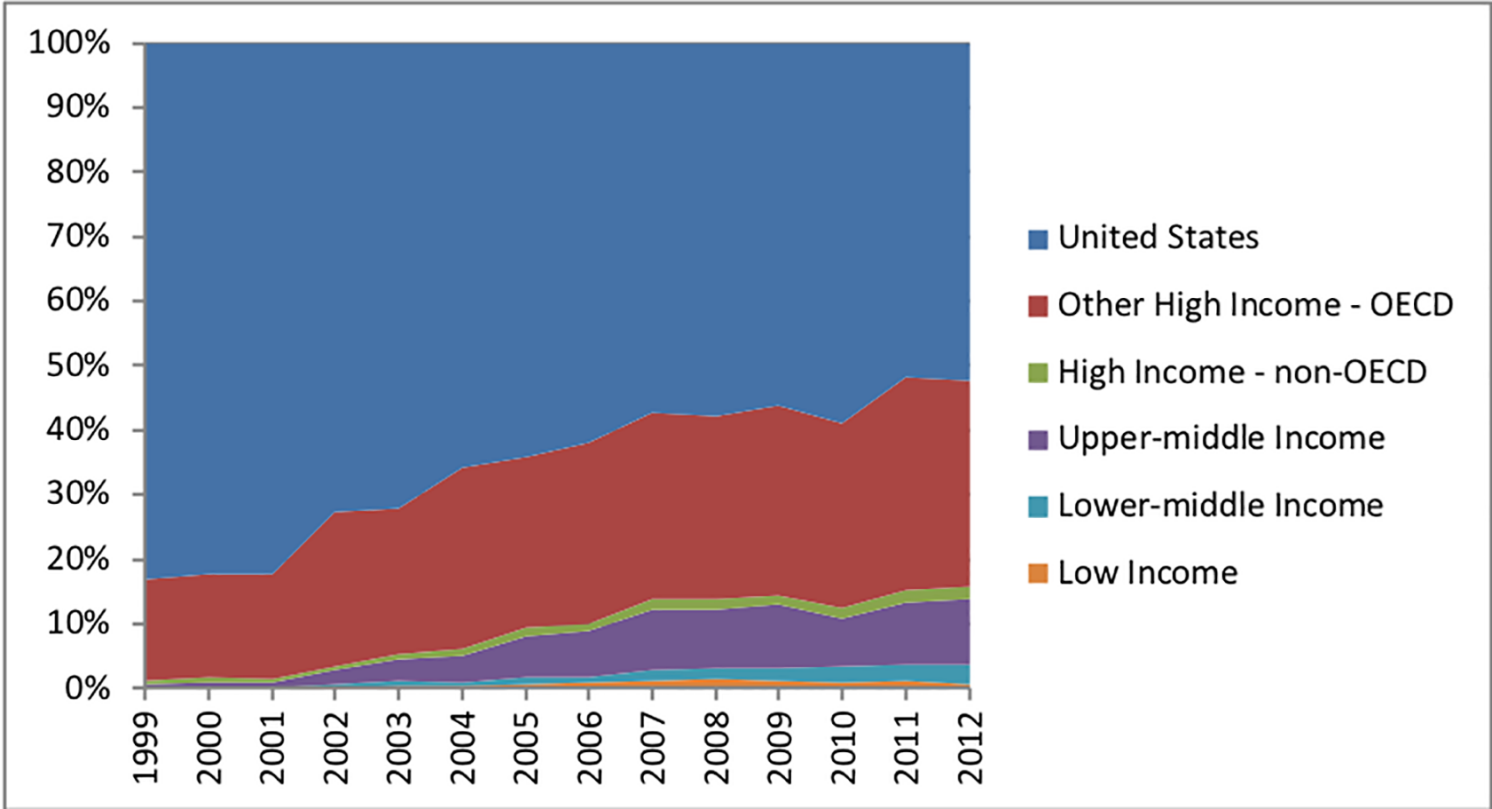
Proportion distribution of registered clinical trials among phase 2 studies by World Bank economic development category. Data for the United States and other high-income OECD countries are displayed separately.

**Fig 5:**
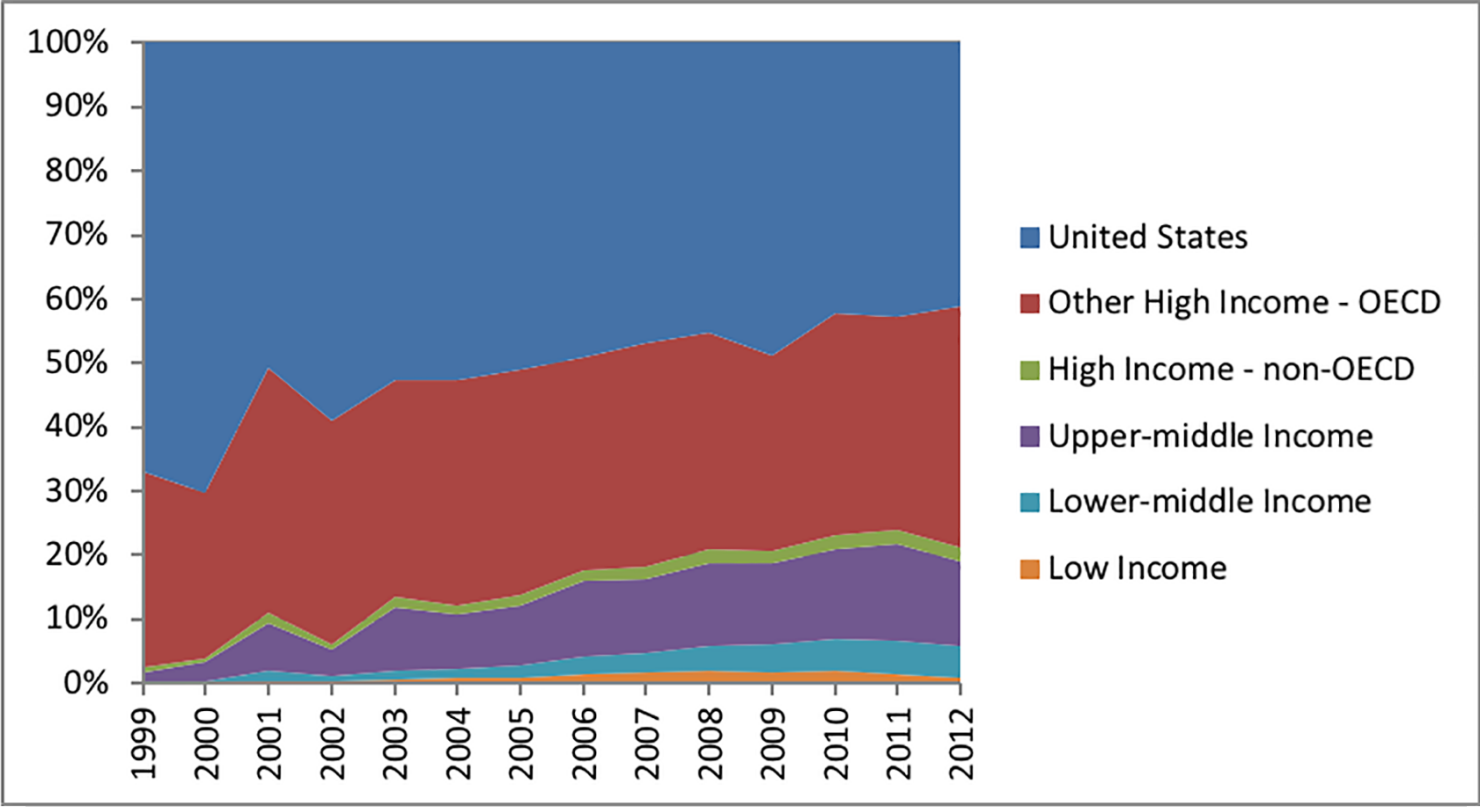
Proportion distribution of registered clinical trials among phase 3 studies by World Bank economic development category. Data for the United States and other high-income OECD countries are displayed separately.

The overall proportion of trial sites remained relatively steady by clinical study phase during the study period. Among studies with an identified phase, 27% were phase 1 trials (12,599 trials), 8% were classified as both phase 1 and phase 2 studies (3,939 trials), 35% were phase 2 studies (16,565 trials), 4% were classified as both phase 2 and 3 studies (2,055 trials), and 26% were phase 3 studies (12,170 trials). When each clinical study phase was stratified by economic development status differences were apparent. High-income OECD countries, including the United States, decreased their proportion of phase 1 clinical trial sites from 93% (year 2006) to 91% (year 2012) (Fig 3). Phase 1 trial sites in countries with emerging economies had risen from 5% (112 trial sites) in 2006 to 7% (267 trial sites) in 2012. The United States and other high-income OECD countries decreased phase 2 trial sites from 90% (year 2006) to 84% (year 2012) (Fig 4). Countries with emerging economies increased their proportion of phase 2 clinical trial sites from 9% (year 2006) to 14% (year 2012). High-income OECD countries, including the United States, decreased their proportion of phase 3 trial sites from 82% (year 2006) to 79% (year 2012) (lowest point was 76% in 2011) (Fig 5). Phase 3 trial sites in countries with emerging economies increased from 16% (8,829 trial sites) in 2006 to 22% (11,555 trial sites) in 2011—declining to 19% (9,468 trial sites) in 2012. Countries with emerging economies (low- and middle-income) operated 19% of phase 3 trial sites, as compared to only 6% of phase 1 trial sites. Countries with emerging economies operated a considerably lower proportion of exploratory phase 1 clinical trials sites, as compared to the United States and other high-income OECD countries. Relative proportional increases for all clinical study phases were highest among upper-middle income and lower-middle income countries.

## Discussion

Using a large global repository of open-access clinical trial data, we demonstrated that the vast majority of clinical trials remain concentrated in high-income countries. Although annual growth rates were highest among low and middle-income countries with emerging economies for both aggregate measures and when stratified by clinical study phase, there remained a relative underrepresentation of phase 1 trials as compared to phase 3 trials in countries with emerging economies. The majority of clinical research was funded by organizations and the pharmaceutical industry; organization-sponsored trials have been increasing. This overall global migration of operational clinical trial sites—particularly for phase 3 trials—to low- and middle-income countries with emerging economies has numerous implications.

These findings are consistent with, but expand upon, several small, geographically limited studies [4,23,24], including our own prior analysis [15]. Thiers et al. used ClinicalTrials.gov data between 2004–2007 to show that most trials were conducted in North America, Western Europe, and Oceania, while growth was occurring in other regions [4]. We expanded on our previous analyses of WHO data [15] by using more comprehensive data based on registration in ClinicalTrials.gov with a longer follow-up period after mandatory registration; by accounting for the number of operational sites within each country and estimated duration of trial site operation; and by evaluating the impact of funding source and clinical study phase [18].

Our findings suggest that a global migration of clinical research is occurring from high-income countries to low and middle-income countries with emerging economies, such as Egypt, Guatemala, China, and Belarus. The relevance for the migration of clinical research is important not only for the protection of human subjects in countries with less regulatory oversight, but also for the translation of clinical research findings to populations with different ethnic and genetic backgrounds. Application of research findings from different populations may have less relevance for clinical effectiveness, and research findings that require additional confirmation would not constitute a cost-effective approach to biomedical research.

Since clinical trials represent approximately 40% of drug development costs [25], it has been suggested that clinical trials may be migrating to resource-limited settings to reduce expenditures [26]. Moving clinical trials to countries with emerging economies can accelerate patient recruitment amidst less regulatory oversight [4,6,7]. As some estimate that roughly 70% of global biomedical research funding is provided by either the United States-based foundations or corporations [27], our data suggest an increase in the outsourcing of clinical research, primarily phase 3 clinical trials, to countries with emerging economies [15]. This expansion is supported by the growth of FDA-regulated clinical investigators based outside the United States [28] and the recent expansion of pharmaceutical companies into Asian markets. This practice raises numerous questions about the translation of clinical trial results to other population, as well as regulatory controls and inspections among governing bodies, including the WHO and FDA.

The small trial density among low-income countries may relate to several factors, including a limited supply of trained clinical researchers. In 2005, when the British Medical Journal launched a themed issue on ‘addressing inequalities in research capacity in Africa’, they received few submissions from African countries [29]. A review of randomized clinical trials conducted on HIV/AIDS in Africa found only 25% included an African principal investigator and most (56%) were funded by agencies outside Africa [30]. Following this, experts agreed that increasing representation of scientists from developing countries was essential for HIV research [31,32]. Since only 1% of recently discovered drugs target neglected tropical diseases [33], addressing global health inequalities may require more clinical scientists to conduct phase 1 trials for the most prevalent conditions in low- and middle-income countries [34]. Furthermore, there are multiple ethical implications about ensuring adequate health care provisions, including providers and hospital beds, to study subjects among all phases of clinical trials, while not occupying already limited health care resources in low- and middle-income countries. At a minimum, all countries should have functioning research ethical review boards in place with community participation and oversight.

Our approach had strengths and limitations. ClinicalTrials.gov is reliant on voluntary registration and subject to underreporting [35]. While >95% of registered clinical trials had complete data in Clinicaltrials.gov after 2005 [14], a period during which there were still changing registration requirements, some trials may have remained unregistered or appeared in other international trial registries, which might bias the observed results [35,17]. Requirements and definitions of trial registration changed during the study period. Registration of phase 1 trials was not required by the ICMJE until 2007 [12]. Further, ICMJE adopted the WHO definition of clinical trials (“any research study that prospectively assigns human participants or groups of humans to one or more health-related interventions to evaluate the effects on health outcomes”) for trials that commenced after June 2008 [12]. Strengths of our analysis included using clinical trial site-years, which attempted to account for the length of time each trial site was operational, and obtaining data at least nine months after the end of 2012 to allow for lags in trial registration. Other strengths were using the most comprehensive global clinical trial registry, assessing trends over a 7-year period, and accounting for the conduct of individual clinical research trials in multiple study locations. In future analyses, smaller, local registries may be queried in order to understand regional patterns of clinical trials. Some more detailed analyses could be performed if clinical trial registries provided data on the individual number of participants enrolled at each clinical trial site.

In conclusion, while clinical trials continue to be concentrated in wealthy countries, the largest percentage growth in registered human clinical research appears to be occurring in countries with emerging economies. This migration of clinical research to emerging economies may be related to expanded training opportunities and/or the high cost of operating clinical trials in high-income countries. Good clinical practices and ethical assurances must be adequate as human clinical research continues to expand, and reporting of clinical trial results should be improved [36]. The geographic expansion of clinical trials requires attention to ensure quality and participant protection, since human participation in clinical research will remain an essential component of advancing medicine.

## Supporting information

S1 Table Country classification by World Bank economic development status. (DOCX)

S2 Table Number of clinical trial site-years by country, ranked by trial site-years 2006–2012. (DOCX)
