# Correction: Global migration of clinical research during the era of trial registration

**DOI:** 10.1371/journal.pone.0199952

**Published:** 2018-06-26

**Authors:** Paul K. Drain, Robert A. Parker, Marion Robine, King K. Holmes, Ingrid V. Bassett

Dr. Ingrid V. Bassett is not included in the author byline. She should be listed as the fifth author and her affiliations are: #4 Medical Practice Evaluation Center, Department of Medicine, Massachusetts General Hospital, Boston, MA, United States of America and #5 Division of Infectious Diseases, Massachusetts General Hospital, Harvard Medical School, Boston, MA, United States of America. The specific contributions of this author are as follows: Conceptualization, Resources, Supervision, and Writing–Review and Editing.

In the Author Contributions section, Robert A. Parker (RAP) should be listed as the author responsible for the paper’s methodology, rather than Paul K. Drain (PKD).

The correct citation is: Drain PK, Parker RA, Robine M, Holmes KK, Bassett IV (2018) Global migration of clinical research during the era of trial registration. PLoS ONE 13(2): e0192413. https://doi.org/10.1371/journal.pone.0192413
