# Supplementary material for: Global migration of clinical research during the era of trial registration
Source: PLoS One. 2018 Feb 28;13(2):e0192413. doi: 10.1371/journal.pone.0192413 (PMC5830297; doi:10.1371/journal.pone.0192413)
Supplement: S1 Table — (DOCX) [file pone.0192413.s001.docx]

S1 Table. Country classification by World Bank economic development status.

| **HIGH income - OECD** |  | **Upper-Middle Income** |  | **Lower-Middle Income** |  | **Low Income** |  |
| --- | --- | --- | --- | --- | --- | --- | --- |
| Australia |  | American Samoa |  | Albania |  | Afghanistan |  |
| Austria |  | Argentina |  | Algeria |  | Bangladesh |  |
| Belgium |  | Belize |  | Angola |  | Benin |  |
| Canada |  | Botswana |  | Armenia |  | Burkina Faso |  |
| Czech Republic |  | Brazil |  | Azerbaijan |  | Burundi |  |
| Denmark |  | Bulgaria |  | Belarus |  | Cambodia |  |
| Finland |  | Chile |  | Bhutan |  | Central African Republic |  |
| France |  | Costa Rica |  | Bolivia |  | Chad |  |
| Germany |  | Croatia |  | Bosnia and Herzegovina |  | Côte d'Ivoire |  |
| Greece |  | Gabon |  | Cameroon |  | The Democratic Republic of the Congo |  |
| Iceland |  | Grenada |  | China |  | Ethiopia |  |
| Ireland |  | Hungary |  | Colombia |  | Gambia |  |
| Italy |  | Kazakhstan |  | Congo |  | Ghana |  |
| Japan |  | Latvia |  | Cuba |  | Guinea |  |
| Korea, Republic of |  | Lebanon |  | Djibouti |  | Guinea-Bissau |  |
| Luxembourg |  | Libyan Arab Jamahiriya |  | Dominican Republic |  | Haiti |  |
| Netherlands |  | Lithuania |  | Ecuador |  | India |  |
| New Zealand |  | Malaysia |  | Egypt |  | Kenya |  |
| Norway |  | Mauritius |  | El Salvador |  | Kyrgyzstan |  |
| Portugal |  | Mexico |  | Fiji |  | Lao People's Democratic Republic |  |
| Spain |  | Northern Mariana Islands |  | Georgia |  | Liberia |  |
| Sweden |  | Oman |  | Guatemala |  | Madagascar |  |
| Switzerland |  | Panama |  | Guyana |  | Malawi |  |
| United Kingdom |  | Poland |  | Honduras |  | Mali |  |
| Unites States |  | Romania |  | Indonesia |  | Mauritania |  |
|  |  | Russian Federation |  | Iran, Islamic Republic of |  | Mongolia |  |
| **High Income - NON-OECD** |  | Saint Kitts and Nevis |  | Iraq |  | Mozambique |  |
| Andorra |  | Serbia |  | Jamaica |  | Myanmar |  |
| Bahamas |  | Montenegro |  | Jordan |  | Nepal |  |
| Bahrain |  | Slovakia |  | Lesotho |  | Niger |  |
| Barbados |  | South Africa |  | Macedonia, FYR |  | Nigeria |  |
| Bermuda |  | Turkey |  | Moldova, Republic of |  | Pakistan |  |
| Brunei Darussalam |  | Uruguay |  | Morocco |  | Papua New Guinea |  |
| Cayman Islands |  | Venezuela |  | Nicaragua |  | Rwanda |  |
| Cyprus |  |  |  | Paraguay |  | Senegal |  |
| Estonia |  |  |  | Peru |  | Sierra Leone |  |
| French Polynesia |  |  |  | Philippines |  | Solomon Islands |  |
| Hong Kong |  |  |  | Sri Lanka |  | Sudan | |
| Israel |  |  |  | Suriname |  | Tanzania | |
| Kuwait |  |  |  | Swaziland |  | Togo | |
| Malta |  |  |  | Syrian Arab Republic |  | Uganda | |
| Monaco |  |  |  | Thailand |  | Uzbekistan | |
| Netherlands Antilles |  |  |  | Tunisia |  | Vietnam | |
| New Caledonia |  |  |  | Ukraine |  | Yemen | |
| Puerto Rico |  |  |  | Vanuatu |  | Zambia | |
| Qatar |  |  |  |  |  | Zimbabwe | |
| Saudi Arabia |  |  |  |  |  |  | |
| Singapore |  |  |  |  |  |  | |
| Slovenia |  |  |  |  |  |  | |
| Taiwan |  |  |  |  |  |  | |
| Trinidad and Tobago |  |  |  |  |  |  | |
| United Arab Emirates |  |  |  |  |  |  | |
| Virgin Islands (U.S.) |  |  |  |  |  |  | |

FYR – Former Yugoslavia Republic of, OECD - Organization for Economic Co-operation and Development.
