# Supplementary material for: Global migration of clinical research during the era of trial registration
Source: PLoS One. 2018 Feb 28;13(2):e0192413. doi: 10.1371/journal.pone.0192413 (PMC5830297; doi:10.1371/journal.pone.0192413)
Supplement: S2 Table — (DOCX) [file pone.0192413.s002.docx]

S2 Table. Number of clinical trial site-years by country, ranked by trial site-years 2006-2012.

| **Rank** | **Country** | **Trial Site-years (2006-2012)** | **Trial Sites (2012)** | **Total Trial Sites (2006-2012)** | **Development  Status** |
| --- | --- | --- | --- | --- | --- |
| **1** | United States | 1,138,447 | 248,591 | 350,592 | OECD |
| **2** | Germany | 190,281 | 40,404 | 57,539 | OECD |
| **3** | France | 134,542 | 26,951 | 52,106 | OECD |
| **4** | Japan | 81,553 | 19,673 | 29,052 | OECD |
| **5** | Canada | 81,379 | 16,525 | 25,380 | OECD |
| **6** | United Kingdom | 61,637 | 13,990 | 19,250 | OECD |
| **7** | Italy | 60,595 | 13,929 | 18,875 | OECD |
| **8** | Spain | 59,281 | 13,576 | 19,770 | OECD |
| **9** | Russian Federation | 38,608 | 8,110 | 12,638 | UMC |
| **10** | Belgium | 37,248 | 7,503 | 11,145 | OECD |
| **11** | Poland | 36,965 | 7,915 | 12,657 | UMC |
| **12** | Australia | 31,424 | 6,949 | 9,852 | OECD |
| **13** | China | 27,932 | 7,797 | 10,681 | LMC |
| **14** | India | 27,287 | 5,329 | 8,753 | LIC |
| **15** | Korea, Republic of | 25,465 | 6,611 | 9,137 | OECD |
| **16** | Netherlands | 25,459 | 5,521 | 8,289 | OECD |
| **17** | Czech Republic | 24,712 | 4,972 | 8,167 | OECD |
| **18** | Brazil | 24,546 | 5,136 | 7,446 | UMC |
| **19** | Hungary | 21,108 | 4,903 | 7,468 | UMC |
| **20** | Israel | 18,560 | 4,198 | 6,333 | N-OECD |

OECD (Organization for Economic Co-operation and Development). OECD – High-income: OECD; N-OECD – High-income: non-OECD; UMC – Upper-Middle Income; LMC – Lower-Middle Income.
